# The iTRAPs: Guardians of Synaptic Vesicle Cargo Retrieval During Endocytosis

**DOI:** 10.3389/fnsyn.2016.00001

**Published:** 2016-02-09

**Authors:** Sarah L. Gordon, Michael A. Cousin

**Affiliations:** ^1^Florey Institute of Neuroscience and Mental Health, The University of MelbourneParkville, VIC, Australia; ^2^Centre for Integrative Physiology, University of EdinburghEdinburgh, UK

**Keywords:** endocytosis, vesicle, clathrin, presynapse, synaptobrevin, synaptotagmin, synaptophysin, SV2A

## Abstract

The reformation of synaptic vesicles (SVs) during endocytosis is essential for the maintenance of neurotransmission in central nerve terminals. Newly formed SVs must be generated with the correct protein cargo in the correct stoichiometry to be functional for exocytosis. Classical clathrin adaptor protein complexes play a key role in sorting and clustering synaptic vesicle cargo in this regard. However it is becoming increasingly apparent that additional “fail-safe” mechanisms exist to ensure the accurate retrieval of essential cargo molecules. For example, the monomeric adaptor proteins AP180/CALM and stonin-2 are required for the efficient retrieval of synaptobrevin II (sybII) and synaptotagmin-1 respectively. Furthermore, recent studies have revealed that sybII and synaptotagmin-1 interact with other SV cargoes to ensure a high fidelity of retrieval. These cargoes are synaptophysin (for sybII) and SV2A (for synaptotagmin-1). In this review, we summarize current knowledge regarding the retrieval mechanisms for both sybII and synaptotagmin-1 during endocytosis. We also define and set criteria for a new functional group of SV molecules that facilitate the retrieval of their interaction partners. We have termed these molecules *i*ntrinsic *tra*fficking *p*artners (iTRAPs) and we discuss how the function of this group impacts on presynaptic performance in both health and disease.

## Introduction

Efficient sustained neurotransmitter release is dependent on the correct reformation of synaptic vesicles (SVs) after stimulation by endocytosis. During this reformation process it is critical that the appropriate cargo molecules are incorporated into SVs with the correct stoichiometry to render SVs functional for the next cycle of neurotransmitter release. During normal physiological stimulation patterns, the majority of cargo are incorporated into SVs during clathrin-mediated endocytosis (CME; Granseth et al., 2006). The selection and clustering of protein cargo during endocytosis is a tightly regulated process, generating SVs that are highly homogenous in both their physical properties and the stoichiometry of their protein content (Takamori et al., 2006; Wilhelm et al., 2014; but see also Mutch et al., 2011). Clathrin adaptor proteins (APs) are central to this process and act as a hub for both SV cargo selection and the recruitment of other accessory endocytosis molecules (Kelly and Owen, 2011). However, when the expression of the classical adaptor molecule AP-2 is reduced using siRNA, or ablated using genomic knockout strategies, relatively minor effects on SV endocytosis at the plasma membrane are observed (Kim and Ryan, 2009; Willox and Royle, 2012; Kononenko et al., 2014; Jung et al., 2015). This suggests that other molecules are required to ensure efficient SV endocytosis and cargo retrieval. In agreement with this, cargo-specific monomeric adaptor molecules have been identified that facilitate the retrieval of cargo that are essential for SV fusion (Rao et al., 2012). Even more intriguing was the revelation that interactions between SV cargo themselves are critical for their accurate and efficient retrieval. This review will summarize progress in this field with particular emphasis on interactions between SV cargoes that facilitate the retrieval of the integral membrane proteins synaptobrevin II (sybII; also known as vesicle-associated membrane protein 2, VAMP2) and synaptotagmin-1. Both sybII and synaptotagmin-1 are indispensable SV cargo molecules, since their genomic deletion results in both the absence of synchronous evoked neurotransmission and early post-natal lethality (Geppert et al., 1994; Schoch et al., 2001). We have termed the SV cargo that interact with these essential proteins as *i*ntrinsic *tra*fficking *p*artners (iTRAPs) and will outline how these trafficking molecules are critical for higher brain function.

## Synaptophysin is an iTRAP for SybII

### Synaptophysin and SybII are Interaction Partners

**Figure 1:**
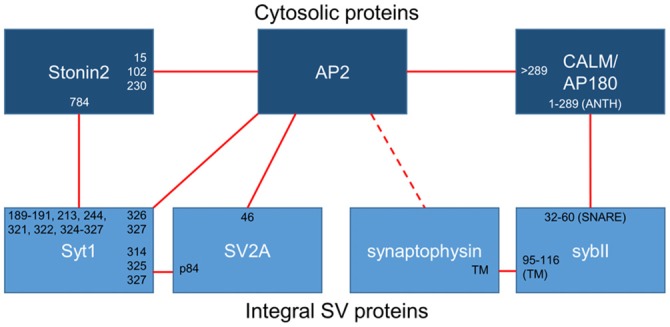
**Binding interactions between essential synaptic vesicle cargo (sybII and synaptotagmin-1; Syt1), iTRAPs (synaptophysin and SV2A), monomeric adaptors (CALM/AP180 and stonin2) and the classical adaptor AP-2.** Cytosolic proteins are dark blue, integral SV proteins are light blue. Solid lines indicate confirmed interactions, dotted lines are putative interactors. The residues (numbers) or domains (ANTH, SNARE or transmembrane; TM) that have been suggested to mediate interactions between these proteins have been noted at the links between partners.

SybII is an essential fusogenic component of the molecular machinery of the SV. It is a vesicular soluble NSF attachment protein receptor (v-SNARE) protein, which, through its association with the plasma membrane target SNAREs syntaxin and SNAP-25 (synaptosomal-associated protein, 25 kDa), renders SVs competent for fusion and drives exocytosis (Jahn and Fasshauer, 2012; Sudhof, 2013). In keeping with its essential role, the correct targeting and localization of sybII to SVs is vital for evoked neurotransmitter release (Schoch et al., 2001). SybII is a single-pass transmembrane SV protein, with a short intraluminal C-terminus and a cytoplasmic N-terminal region that contains a highly conserved SNARE motif (Sudhof et al., 1989), with the extreme N-terminus being involved in protein-protein interactions (Martincic et al., 1997; Burré et al., 2010). It is the most abundant cargo on the SV with approximately 70 copies (Takamori et al., 2006; Wilhelm et al., 2014), however it is estimated that only 1–3 sybII molecules are required for membrane fusion (Domanska et al., 2009; Mohrmann et al., 2010; van den Bogaart et al., 2010; Sinha et al., 2011). Synaptophysin is the second most abundant cargo on the SV, with approximately 30 copies present (Takamori et al., 2006; Wilhelm et al., 2014). It contains four transmembrane domain regions and cytoplasmic N- and C-termini, the latter being the major site for protein—protein interactions (Daly and Ziff, 2002; Wheeler et al., 2002; Felkl and Leube, 2008). Synaptophysin shares an interaction with sybII (Calakos and Scheller, 1994; Edelmann et al., 1995; Washbourne et al., 1995), thus the two most abundant cargoes on the SV also interact together (Figure 1).

The exact domains that participate in the synaptophysin-sybII interaction remain to be elucidated despite the fact that this association was one of the first to be identified at the presynapse. Several studies have suggested that the proteins interact through their transmembrane domains (Becher et al., 1999; Yelamanchili et al., 2005; Bonanomi et al., 2007), with the C-terminal portion of the sybII transmembrane domain being key for this interaction (Yelamanchili et al., 2005). Single particle electron microscopy analysis of the native mammalian synaptophysin—sybII complex revealed that the transmembrane domains of synaptophysin and sybII interact in a 6:12 ratio, with synaptophysin forming a hexameric core (Thomas et al., 1988; Arthur and Stowell, 2007) that binds six sybII dimers (Adams et al., 2015). Thus it is possible that the synaptophysin—sybII complex is a multimeric structure that clusters 12 sybII molecules per functional unit.

While the core interaction site for sybII and synaptophysin is likely to be the transmembrane domains of the proteins, other factors may regulate their association. For example, specific membrane lipids are proposed to contribute to the strength of synaptophysin-sybII interaction, with increases in cholesterol content favoring the binding of synaptophysin to sybII (Mitter et al., 2003; Adams et al., 2015). The synaptophysin-sybII interaction is also upregulated during mammalian development, and addition of adult cytosolic extract to SVs isolated from embryos increases the prevalence of the synaptophysin-sybII complex, suggesting a cytosolic protein regulates the interaction (Becher et al., 1999). In agreement, the addition of a peptide encompassing the N-terminal 32 amino acids of sybII dissociates the synaptophysin-sybII complex *in vitro* (Washbourne et al., 1995). Thus the abundant SV cargoes synaptophysin and sybII directly interact to form a large multimeric structure, which may be regulated by the local membrane and cytosolic environment.

The functional role of the synaptophysin-sybII interaction has been a source of intense scrutiny. SybII binds in a mutually exclusive manner to either synaptophysin or the plasma membrane t-SNAREs (Edelmann et al., 1995; Siddiqui et al., 2007), suggesting that it controls access of sybII to the SNARE complex. In support, an activity-dependent dissociation of the synaptophysin-sybII complex occurs following incubation with α-latrotoxin by both forster resonance energy transfer (FRET) analysis of exogenously expressed proteins in neurons (Pennuto et al., 2003) and immunoprecipitation from isolated nerve terminals (Reisinger et al., 2004). Interestingly calcium is required, though not sufficient, for this dissociation in intact nerve terminals (Chapman et al., 1995; Prekeris and Terrian, 1997; Daly and Ziff, 2002; Reisinger et al., 2004). However, synaptophysin readily dissociates from sybII in the presence of the t-SNAREs (Siddiqui et al., 2007), arguing against the proposed role as modulator of SNARE complex formation. Moreover, the synaptophysin-sybII interaction is upregulated following membrane fusion (Khvotchev and Sudhof, 2004), suggesting a functional role post-fusion for this interaction between the two major SV proteins.

### Synaptophysin is Essential for Accurate SybII Retrieval during Endocytosis

The majority of the studies discussed above examined the formation and dissociation of the synaptophysin-sybII complex in isolation from its location within the nerve terminal. Fluorescence microscopy studies using genetically-encoded reporters have provided key insights into the physiological role of the synaptophysin-sybII interaction as a regulator of sybII localization *in situ*. When exogenous sybII was overexpressed in either heterologous expression systems or cultured neurons, it exhibited a predominantly plasma membrane localization (Pennuto et al., 2003; Bonanomi et al., 2007), suggesting it was being inefficiently targeted to vesicular compartments. The targeting of SV cargo occurs principally during CME in central nerve terminals, with AP-2 performing a key role (Kelly and Owen, 2011). However sybII lacks classical recognition motifs for AP-2, suggesting that it was targeted to SVs via a non-canonical mechanism. Interestingly when synaptophysin is co-expressed with sybII in similar overexpression studies, the latter is efficiently redirected to SVs (Pennuto et al., 2003; Bonanomi et al., 2007).

Recent studies in cultured neurons from synaptophysin knockout mice have confirmed that synaptophysin is critical for sybII targeting to SVs. In these studies endogenous sybII was mislocalized from nerve terminals, and exogenous sybII accumulated at the plasma membrane (Gordon et al., 2011). Crucially, this mistargeting is a direct result of a specific deficit in the activity-dependent retrieval of sybII during compensatory endocytosis, with sybII retrieval kinetics being severely slowed in synaptophysin knockout neurons. The efficient retrieval of sybII was fully rescued by the re-addition of wild-type synaptophysin back into these null neurons (Gordon et al., 2011). Confirmation of a key role for synaptophysin in sybII retrieval came from analysis of a series of synaptophysin mutants identified in X-linked intellectual disability. Every synaptophysin mutant tested failed to restore normal sybII retrieval in this knockout model system (Gordon and Cousin, 2013). Notably, all but one of these mutations are predicted to ablate or interfere with the transmembrane synaptophysin-sybII interaction (Adams et al., 2015). The final mutation causes substantial changes in the cytosolic tail of synaptophysin which, as outlined above, may regulate the strength of the interaction. Together, this data establishes that synaptophysin is essential for the accurate retrieval of sybII during endocytosis and as such is the founding member is the iTRAP group of SV proteins.

### Monomeric Adaptor Protein AP180/CALM Facilitates SybII Retrieval

Synaptophysin is necessary for the accurate retrieval of sybII during endocytosis, however there is an equal requirement for other endocytosis molecules. As stated above, sybII has no canonical recognition motifs for AP-2; therefore another molecule must be present to recruit sybII to the endocytosis machinery. Synaptophysin may contribute to this role, since it contains a series of tyrosine-based repeats that may be recognized by the μ2 subunit of AP-2 (Sudhof et al., 1987). The likely molecular bridge between sybII and AP-2 however is the monomeric clathrin adaptor protein AP180 and the related protein clathrin assembly lymphoid myeloid leukemia (CALM; Figure 1). Both AP180 and CALM are highly conserved throughout evolution; they bind to both AP-2 and clathrin to facilitate endocytosis, and they both interact with sybII to mediate its retrieval (Koo et al., 2011b).

AP180/CALM was originally identified as a regulator of sybII targeting to synapses in *Caenorhabditis elegans.* Mutations in *unc11* (the *C. elegans* homolog of AP180 and CALM) resulted in reduced targeting of sybII to the neuropil and a broad distribution of sybII throughout the neuron (Nonet et al., 1999). Further work revealed that sybII mislocalization was a common feature of several AP180/CALM knockout systems. For example sybII is mislocalized along the axon in *lap* (*Drosophila* homolog of AP180) mutant flies (Bao et al., 2005) and in CALM/AP180-depleted mammalian neuronal cultures (Koo et al., 2011a). In addition sybII is both stranded on the plasma membrane and inefficiently retrieved during endocytosis in AP180 knockout neurons (Koo et al., 2015). Knockdown of CALM in AP180 knockout neurons exacerbates the surface stranding of sybII (Koo et al., 2015). In mammalian systems this mislocalization phenotype is specific to sybII, however in either *lap* null flies (Bao et al., 2005) or in flies where *lap* was acutely inactivated by fluorescein-assisted light inactivation (Vanlandingham et al., 2014), several other presynaptic proteins (including synaptotagmin, vGLUT and cysteine-string protein) were also mislocalized along axons in conjunction with sybII. Further work is therefore required to delineate the specificity of AP180/CALM as a modulator of sybII retrieval vs. its actions as a regulator of CME.

The interaction site between AP180/CALM and sybII has been mapped, with the ANTH (AP180 N-terminal homology) domains of either AP180 or CALM binding to the SNARE domain of sybII (Koo et al., 2011a; Miller et al., 2011). When this interaction was perturbed in primary neurons by either point mutations in the SNARE motif of sybII or in the ANTH domain of AP180, exogenously expressed sybII is mislocalized to the cell surface (Koo et al., 2011a, 2015), confirming the requisite nature of this interaction in sybII retrieval. Thus in addition to the iTRAP synaptophysin, AP180/CALM is also essential for the accurate retrieval of sybII during SV endocytosis.

**Figure 2:**
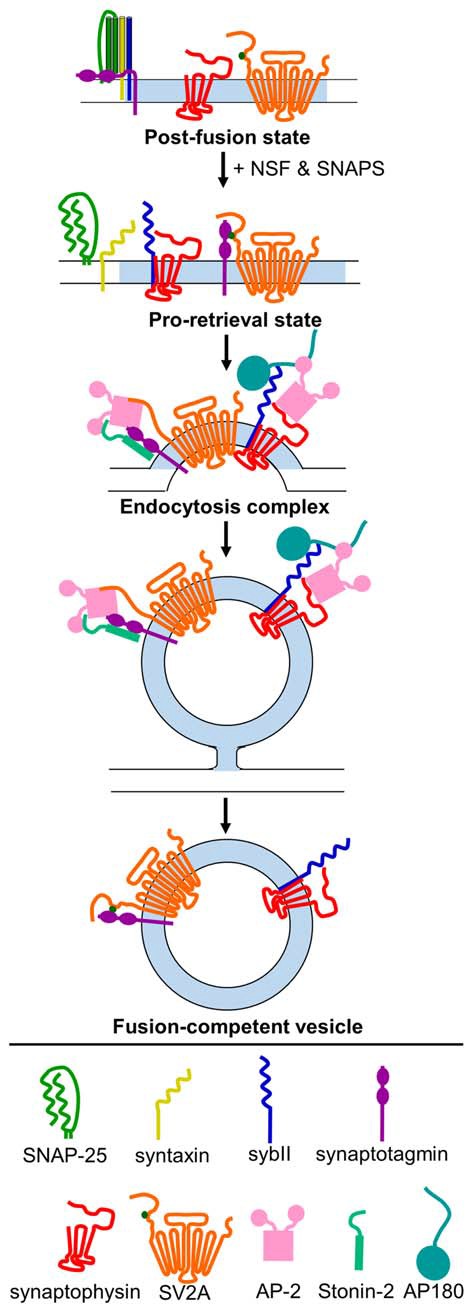
**Model for mechanism of retrieval of essential SV cargo.** Following exocytosis (post-fusion state), sybII (navy blue) is trapped in a cis-SNARE complex with SNAP-25 (green) and syntaxin (yellow), and the C2A and C2B domains of synaptotagmin-1 (purple) are embedded in the membrane. N-ethylmaleimide-sensitive factor (NSF) and soluble NSF-attachment proteins (SNAPs) act to dissociate the cis-SNARE complex, freeing sybII in the plasma membrane. Synaptophysin (red) binds to sybII to protect it from re-entering into futile cis-SNARE complexes, and T84-phosphorylated (dark green dot) SV2A (orange) binds to synaptotagmin-1 to create a pro-retrieval state. This allows AP180/CALM (dark teal) to bind to the SNARE-domain of sybII. AP-2 (pink) binds to AP180/CALM, and may also interact with synaptophysin. Simultaneously, SV2A (now dephosphorylated) dissociates from synaptotagmin-1 and binds to AP-2, which strengthens the binding of AP-2 to synaptotagmin-1. Stonin-2 (light teal) also binds to the C2 domains of synaptotagmin-1 and to AP-2 to strengthen the interaction. Together, this promotes the clustering of these cargoes for endocytosis. Following endocytosis, the monomeric adaptors AP180/CALM and stonin 2, and the classical clathrin adaptor AP-2, dissociate from their vesicle binding partners. Synaptophysin remains bound to sybII, and rephosphoryated SV2A binds to synaptotagmin-1 to produce a fusion-competent vesicle.

As the studies above indicate, the trafficking of sybII during SV turnover requires exquisite precision, and if this process is disrupted a range of deleterious effects emerge that impact on neuronal function. The dual requirement for both synaptophysin and AP180/CALM indicates that both are essential for accurate sybII trafficking, however it is still unclear how and whether they cooperate to do so. Both interact with sybII at mutually exclusive sites (Figure 1), and we have proposed that synaptophysin may restrict the entry of sybII into futile plasma membrane cis-SNARE complexes to allow AP180 access to its SNARE motif (Gordon and Cousin, 2014; Figure 2). Future experiments examining how ablation of the expression of both synaptophysin and AP180/CALM impacts on sybII trafficking should provide valuable insights into how these molecules co-operate at the synapse in this key role.

## SV2A is a Phospho-Dependent iTRAP for Synaptotagmin-1

### SV2A Binds to Synaptotagmin-1 in a Phosphorylation-Dependent Manner

The demonstration that synaptophysin is required for the accurate retrieval of sybII suggests that other potential SV cargo interactions may nucleate and facilitate their own retrieval. In agreement a second iTRAP relationship has been identified, with synaptic vesicle protein 2A (SV2A) being essential for the accurate retrieval of the calcium sensor synaptotagmin-1.

SV2A is ubiquitously expressed in the central nervous system, whereas other members of the larger gene family, are either brain-specific (SV2B) or a minor isoform (SV2C; Buckley and Kelly, 1985; Bajjalieh et al., 1994). SV2A has a requisite role in postnatal brain function, since knockout mice fail to grow and experience severe seizures before premature death after 3 weeks (Crowder et al., 1999; Janz et al., 1999a). The presynaptic role of SV2A is still unclear however, with most research focussing on a potential function as a calcium-dependent effector of SV exocytosis (Mendoza-Torreblanca et al., 2013). These potential roles include the control of short term synaptic plasticity either via regulation of residual intracellular calcium (Janz et al., 1999a; Chang and Südhof, 2009; Wan et al., 2010) or the size of the readily releasable pool (Custer et al., 2006). In contrast, the role for synaptotagmin-1 in presynaptic function is well established. It is essential for coupling calcium influx to evoked synchronous neurotransmitter release by binding calcium via two low affinity C2 domains (Brose et al., 1992; Geppert et al., 1994). Calcium binding neutralizes local negative charge on the C2 domains, allowing hydrophobic residues to enter the plasma membrane and evoke SV fusion (Bai et al., 2002; Martens et al., 2007; Hui et al., 2009).

The sites for the SV2A—synaptotagmin-1 interaction have been mapped, with the N-terminus of SV2A required for binding to the C2B domain of synaptotagmin-1 (Schivell et al., 1996, 2005). Phosphorylation of the N-terminus of SV2A by casein kinase 1 family kinases strongly potentiates this interaction (Pyle et al., 2000; Zhang et al., 2015). The *in vivo* phosphorylation sites on SV2A were recently identified and phosphorylation of one specific residue within the N-terminus (Thr84) permitted binding to synaptotagmin-1. The interaction site for phospho-SV2A was a basic patch of amino acids on the C2B domain (Zhang et al., 2015; Figure 1) which overlaps with interaction sites for a series of other molecules including phosphoinositides (Schiavo et al., 1996), calcium channels (Leveque et al., 1992), t-SNARE dimers (Bhalla et al., 2006) and even other synaptotagmins (Chapman et al., 1996). This suggests that competition may exist for binding to this region within the C2B domain, even though the combination of lysines bound by phospho-SV2A is thus far unique. It will therefore be of high importance to delineate how other molecules compete with SV2A for synaptotagmin-1 binding during SV endocytosis.

### SV2A Acts as an iTRAP to Direct Synaptotagmin-1 Retrieval to SVs

SV2A not only binds synaptotagmin-1, but functions as an iTRAP to direct synaptotagmin-1 targeting to SVs. Endogenous or exogenously expressed synaptotagmin-1 accumulates at the plasma membrane in either SV2A knockout neurons (Yao et al., 2010) or in neurons depleted of SV2A (Kaempf et al., 2015; Zhang et al., 2015). This effect was SV2A-dependent, since normal plasma membrane synaptotagmin-1 levels were restored by expression of the wild-type protein in either knockout or knockdown neurons (Yao et al., 2010; Kaempf et al., 2015; Zhang et al., 2015). This effect is also specific for synaptotagmin-1, since other SV cargoes do not accumulate under the same experimental conditions (Kaempf et al., 2015). Importantly, synaptotagmin-1 remained stranded on the plasma membrane when knockout/knockdown neurons were rescued with mutant SV2A that was deficient for either SV2A internalization (Yao et al., 2010) or synaptotagmin-1 binding (Zhang et al., 2015). This indicates that an interaction with SV2A was required for the efficient retrieval of synaptotagmin-1 from the plasma membrane. In support the number of synaptotagmin-1 molecules that visited the plasma membrane during an action potential train (i.e., that were present on a SV) was reduced in the absence of SV2A (Kaempf et al., 2015).

In addition to plasma membrane accumulation, synaptotagmin-1 retrieval during SV endocytosis was accelerated in the absence of SV2A (Kaempf et al., 2015; Zhang et al., 2015). This was not a general acceleration of endocytosis, since other SV cargo were not affected in a similar manner (Yao et al., 2010; Zhang et al., 2015; but see Kaempf et al., 2015). Therefore the absence of SV2A results in synaptotagmin-1 accumulation at the plasma membrane, but also an acceleration of its retrieval. This was an unusual finding, since increased SV cargo stranding at the plasma membrane is usually a surrogate for perturbed retrieval by endocytosis (Voglmaier et al., 2006; Kim and Ryan, 2009; Yao et al., 2010; Koo et al., 2011a; Willox and Royle, 2012; Foss et al., 2013; Kononenko et al., 2014) such as described above for sybII in synaptophysin or AP180 knockout neurons (Gordon et al., 2011; Koo et al., 2015). There are a number of potential explanations for this phenotype. First, the lack of an SV2A interaction may result in synaptotagmin-1 being retrieved by a parallel endocytic mode with faster retrieval kinetics than classical CME. Second, it may be that retrieval of synaptotagmin-1 during spontaneous SV recycling is reduced (reflected in increased surface accumulation in resting neurons), but activity-dependent retrieval is facilitated. Arguing against this is the finding that normal plasma membrane synaptotagmin-1 levels are restored when neuronal activity is silenced in SV2A knockdown neurons (Kaempf et al., 2015). It will be critical to resolve these seemingly contradictory observations to determine the molecular role for this interaction in synaptotagmin-1 retrieval.

### Stonin-2 and AP-2 are also Required for Efficient Synaptotagmin-1 Retrieval

The iTRAP SV2A is not the only molecule required for accurate synaptotagmin-1 retrieval during SV endocytosis. The C2B domain of synaptotagmin-1 has a well-established interaction with the μ2 subunit of AP-2 within the same patch of basic residues required for SV2A binding (Zhang et al., 1994; Chapman et al., 1998; Haucke et al., 2000; Figure 1). C2B domain multimerization may also increase synaptotagmin-1 affinity for AP-2 (Grass et al., 2004). Interestingly short peptides from SV2A (that contain a canonical tyrosine-based endocytosis motif) increase the amount of AP-2 extracted from brain cytosol by the C2B domain of synaptotagmin-1 (Haucke and De Camilli, 1999; Haucke et al., 2000; Grass et al., 2004), suggesting that SV2A binding alters AP-2 in such a way that it allosterically promotes its binding to synaptotagmin-1. In support of this concept, the interaction site for this canonical motif within the μ2 subunit of AP-2 is distinct from the region that interacts with the C2B domain, explaining its ability to increase affinity (Haucke et al., 2000). Co-immunoprecipitation studies have also shown the presence of a tripartite complex of SV2A, AP-2 and synaptotagmin-1, suggesting they form a retrieval complex in mammalian brain (Haucke and De Camilli, 1999). Thus a potential iTRAP function of SV2A may be to facilitate synaptotagmin-1 interactions with the key endocytosis adaptor complex AP-2.

Efficient synaptotagmin-1 retrieval also requires the monomeric adaptor protein stonin-2. The interaction with stonin-2 is distributed over both C2 domains of synaptotagmin-1, however stonin-2 primarily interacts with the C2A domain (Jung et al., 2007; Figure 1). Very close parallels exist between stonin-2 and SV2A with respect to synaptotagmin-1 trafficking. In stonin-2 knockout neurons synaptotagmin-1 accumulates at the plasma membrane and displays an accelerated retrieval during SV endocytosis, in an identical manner to that seen in the absence of SV2A (Kononenko et al., 2013). However stonin-2 knockout neurons also display an acceleration of SV endocytosis, something not observed in neurons lacking SV2A. The similar phenotype in neurons lacking either SV2A or stonin-2 suggests that these molecules may be functionally redundant for synaptotagmin-1 trafficking. This was recently tested by silencing SV2A expression in stonin-2 knockout neurons either by genomic knockout or siRNA (Kaempf et al., 2015). This work revealed an exacerbation of plasma membrane synaptotagmin-1 accumulation and a further acceleration of its retrieval, suggesting both stonin-2 and SV2A are required for efficient synaptotagmin-1 trafficking. In addition, this suggests that SV2A and stonin-2 either act at discrete parallel steps or alternatively perform parallel additive roles in synaptotagmin-1 retrieval.

At least three molecules are required for efficient synaptotagmin-1 retrieval, however how do SV2A, stonin-2 and AP-2 coordinate this event during neuronal activity? The key may be the fact that almost all members share interactions with each other (Figure 1), ensuring maximum efficiency when all are present and redundancy when one is absent or mutated. In support of a potential redundancy of action, the retrieval kinetics of synaptotagmin-1 that has either C2A or C2B deleted are indistinguishable from wild-type, however these mutants did display defective targeting to nerve terminals (Yao et al., 2012a). Both stonin-2 and SV2A can interact with AP-2 via canonical tyrosine-based internalization motifs (Diril et al., 2006; Yao et al., 2010). As stated above, the binding of these canonical peptide motifs to AP-2 increases the affinity of the synaptotagmin-1 C2B domain for AP-2 (Haucke and De Camilli, 1999). In addition, both stonin-2, and either SV2A or AP-2, should be able to access the C2A and C2B domains of synaptotagmin-1 simultaneously, based on the interaction sites between the proteins (Figure 1). One key regulatory step may be the phosphorylation of SV2A, since this may determine priority of access for the C2B domain between itself and AP-2 (for model, see Figure 2). It may be that phosphorylated SV2A binds to the C2B domain of synaptotagmin-1 and acts as a target for AP-2 recruitment. Upon binding to AP-2, SV2A dissociates from synaptotagmin-1 (perhaps facilitated by dephosphorylation of T84) and potentiates the binding of AP-2 to the C2B domain. This interaction also increases the association of AP-2 with the membrane (Haucke and De Camilli, 1999) and facilitates endocytosis. Stonin-2 continues to interact with synaptotagmin-1 through the C2A and C2B domains, as well as binding to AP-2, to further assist in synaptotagmin-1 retrieval and endocytosis. It will therefore be of great future interest to determine where (on SVs and/or plasma membrane) and when (before, during or after action potential stimulation) SV2A is phosphorylated by casein kinase1 family kinases or dephosphorylated by an as yet unidentified phosphatase.

## Common Themes in SV Cargo Retrieval: Do Other iTRAPs Exist?

Evidence is emerging that SV cargo retrieval during endocytosis contains a number of “fail-safe” mechanisms that ensure SVs are formed with the correct molecules with the required stoichiometry. Two of the most important cargoes on SVs are sybII and synaptotagmin-1, which are both essential for synchronous calcium-dependent SV fusion. It is becoming apparent that mammalian nerve terminals contain a high level of functional redundancy to ensure both are retrieved with high fidelity.

There are large similarities between the properties and retrieval mechanisms for both sybII and synaptotagmin-1. First, both of these essential cargoes are single-pass transmembrane proteins. Second, their SV interaction partners, the iTRAPs (synaptophysin and SV2A), are multiple pass transmembrane proteins that have arisen relatively late in evolution, only being present in vertebrate species at a functional level. Third, both sybII and synaptotagmin-1 require a monomeric adaptor protein (AP180/CALM for sybII and stonin-2 for synaptotagmin-1) for efficient retrieval. Finally the classical adaptor protein complex AP-2 is also required. Removal of any of these components results in perturbed cargo retrieval. In addition both synaptophysin and SV2A are heavily glycosylated, which is important for their targeting to SVs (Kwon and Chapman, 2012). We predict that this will also impact on the retrieval of their specific retrieval partner, however this remains to be tested experimentally.

Another similarity between sybII and synaptotagmin-1 is that they are both proposed to be required for efficient SV endocytosis in their own right. For example, SV endocytosis is slowed when sybII is depleted from neurons either at the genomic level (Deák et al., 2004), using knockdown with shRNA (Zhang et al., 2013) or after incubation with the clostridial neurotoxin tetanus toxin (Hosoi et al., 2009; Xu et al., 2013). Similarly, multiple studies have shown that reduction or ablation of synaptotagmin-1 expression retards SV retrieval kinetics in both invertebrate and mammalian systems (Jorgensen et al., 1995; Poskanzer et al., 2003; Yao et al., 2012a,b). It will be critical to determine whether these potential roles in endocytosis are functionally distinct from their interaction with iTRAPs, monomeric adaptors and AP-2.

The identification of cognate iTRAPs that control the accurate retrieval of both sybII and synaptotagmin-1 lead to possibility that other key interactions may occur between SV cargo to facilitate their SV targeting and retrieval. The similarities between the iTRAPS, and their later appearance through evolution, suggest that common rules drive their function. Other candidate proteins that fit at least some of the criteria above include the synaptophysin-related tetraspanin proteins, synaptoporin and synaptogyrin. Synaptoporin is an N-glycosylated protein (Fykse et al., 1993) that can also bind sybII (Edelmann et al., 1995; Becher et al., 1999), however the functional role of this interaction has not yet been investigated. Unlike synaptophysin, synaptoporin has a restricted expression in the mammalian central nervous system (Marquèze-Pouey et al., 1991; Fykse et al., 1993) and displays a different expression pattern during development (Marquèze-Pouey et al., 1991), suggesting that it may play a role in specific neuronal populations. Intriguingly, the phenotype in synaptophysin null neurons that also naturally lack synaptoporin is more severe than in other neuronal groups (Spiwoks-Becker et al., 2001); whether deficient sybII retrieval underlies this phenotype is unknown. In synaptogyrin mutant *C. elegans*, sybII displayed a more diffuse localization, with wider puncta than in wild-type worms, suggesting a deficit in sybII recruitment to nerve terminals (Abraham et al., 2011). Synaptogyrin is the major vesicular tetraspanin in the *C. elegans* nervous system (Abraham et al., 2006) and may thus be acting to functionally replace synaptophysin in worms. In addition, mice lacking both synaptophysin and synaptogyrin displayed synaptic dysfunction that was not evident in single knockout mice, with deficits in both paired-pulse facilitation and long-term potentiation (Janz et al., 1999b). Together this data provides tantalizing evidence that synaptoporin and synaptogyrin may play neuronal subtype- or species- specific roles as iTRAPs, to either support or reproduce the function of synaptophysin in sybII retrieval, however further studies are required to definitively address this.

It is possible that other potential iTRAPs conform to only a subset of the relatively stringent criteria we have outlined above for the trafficking of both sybII and synaptotagmin-1. In agreement, depletion of the SV glutamate transporter (vGLUT) has disparate effects on the retrieval of a subset of SV cargo, including sybII, SV2A and synaptophysin, but not synaptotagmin-1 (Pan et al., 2015). The retrieval of these cargoes was proposed to be coordinated by a C-terminal proline-rich motif on vGLUT. However, previous work examining C-terminal interaction partners did not demonstrate binding to SV cargo, but instead identified interactions with a subset of src-homology 3 domain containing proteins and E3 ubiquitin ligases (Santos et al., 2014). Furthermore phospho-mimetic or -null substitutions on the vGLUT C-terminal tail altered AP-2 interactions (Santos et al., 2014), suggesting that this region is important for directing its own retrieval to SVs. Therefore vGLUT is a third putative member of the iTRAP group, however further work is required to confirm its interaction with SV proteins, and determine the molecular mechanisms underlying its potential coordination of SV cargo retrieval.

## Dysfunction of the iTRAPs and Human Disease

Maintenance of robust, efficient retrieval of core SV protein cargo within the brain is central to human health. The iTRAPs therefore play a crucial role in correct brain functioning throughout an individual’s lifetime. As evidence for this, alterations in either iTRAP levels or functionality have been implicated in a spectrum of neurological conditions, from congenital, early-onset neurodevelopmental disorders through to neurodegenerative diseases that arise in later life.

### Dysfunctional iTRAP Trafficking of SybII in Human Disease

Mutations in the iTRAP synaptophysin have been identified in individuals with familial forms of intellectual disability (Tarpey et al., 2009). Concordantly, synaptophysin KO mice have mild deficits in learning and memory (Schmitt et al., 2009). Importantly, each of the intellectual disability-associated variants of synaptophysin have a reduced ability to coordinate sybII retrieval during endocytosis (Gordon and Cousin, 2013), suggesting that is it specifically this process that is perturbed in individuals harboring mutations in synaptophysin. Synaptophysin has also been implicated in schizophrenia, with multiple rare variants identified that were absent in control patients (Shen et al., 2012). Dysfunctional sybII retrieval may also alter excitatory/inhibitory balance (see below) and in agreement, the interaction between synaptophysin and sybII is upregulated following epileptic seizures in the kindling rat model of epilepsy (Hinz et al., 2001). Therefore the perturbation or loss of synaptophysin function has been linked to series of neurodevelopmental disorders that are associated with deficits in higher brain function. This suggests that loss of fine control of sybII retrieval impacts on the complex circuitry required for correct cognitive function in humans.

Further evidence that dysfunctional sybII trafficking impacts on excitatory/inhibitory balance originate from AP180 knockout mice, which die prematurely as a result of seizure activity (Koo et al., 2015). Interestingly, a more severe sybII trafficking defect was observed in inhibitory interneurons from these mice. Silencing activity in these neurons restored sybII localization to wild-type levels, suggesting the higher background activity of inhibitory interneurons (Bartos et al., 2007) exacerbated sybII retrieval defects and potentially precipitated seizure activity (Koo et al., 2015).

In addition to neurodevelopmental disorders, synaptophysin dysfunction is also linked to neurodegenerative conditions such as Alzheimer’s disease (AD). Intriguingly, synaptophysin levels were reduced in the earliest stages of AD (mild AD) whilst there was no alteration in other presynaptic molecules such as synaptotagmin-1 or GAP-43, suggesting that early in disease progression there may be a selective reduction in synaptophysin (Masliah et al., 2001). A reduction in sybII levels mimics the decline in synaptophysin expression in AD, again without an appreciable loss of synaptotagmin-1 (Shimohama et al., 1997) suggesting these two SV molecules are functionally linked. This raises questions regarding the mechanisms by which synaptophysin and sybII are selectively affected in AD, and at which stage in an individual’s life these levels are altered. Interestingly, one of the peptides linked to AD pathogenesis, Aβ42, binds to synaptophysin, and modulates the interaction between synaptophysin and sybII (Russell et al., 2012). Soluble Aβ levels correlate with pathogenic markers of AD progression (McLean et al., 1999), and impair synaptic function (Selkoe, 2008; Mucke and Selkoe, 2012). Disruption of the synaptophysin-sybII interaction by Aβ42 may therefore result in perturbed sybII trafficking in AD, and perhaps underlie early synaptic dysfunction and ultimately lead to synapse and neuronal loss later in disease progression.

AP180/CALM have also been linked to AD through genome-wide association studies (Harold et al., 2009). In addition CALM is abnormally cleaved in AD and found associated with neurofibrillary tangles, which are intraneuronal aggregates of hyperphosphorylated tau (Ando et al., 2013). Interestingly, CALM also regulates tau clearance through v-SNARE dependent modulation of autophagy (Moreau et al., 2014), with AP180 regulating generation of toxic Aβ peptides (Wu et al., 2009). How any of these proposed pathological functions relates to sybII trafficking and retrieval is unknown, and requires further investigation.

### Dysfunctional iTRAP Trafficking of Synaptotagmin-1 in Human Disease

Dysfunction of the other iTRAP SV2A has been strongly linked to epilepsy. SV2A knockout mice have severe epileptic seizures, fail to grow, and die by 3 weeks old (Crowder et al., 1999; Janz et al., 1999a). Remarkably, SV2A is also the binding partner for the leading anti-epileptic drug, levetiracetam (Lynch et al., 2004). The mechanism by which levetiracetam exerts its effect is still under debate, but its potency directly relates to SV2A binding; the binding affinity of SV2A ligands is proportional to their protective effects in multiple models of epilepsy (Lynch et al., 2004; Kaminski et al., 2008). Furthermore mice heterozygous for SV2A knockout have higher incidence of spontaneous seizures compared to wild-type mice (Crowder et al., 1999), a reduced threshold for induced seizure activity, and a reduced responsiveness to levetiracetam (Kaminski et al., 2009). It is still unclear whether levetiracetam modulates SV2A function or whether it simply uses SV2A as an activity-dependent carrier to gain access to the presynapse. The latter possibility is supported by studies showing that levetiracetam binds to a series of residues in the transmembrane domains of SV2A (Correa-Basurto et al., 2015), with D670 in the tenth transmembrane domain being identified as a key residue (Lee et al., 2015). Furthermore functional studies have shown that the time for levetiracetam to reduce evoked SV recycling and neurotransmitter release is shortened greatly by prior neuronal activity (Meehan et al., 2011, 2012). Evidence also exists that levetiracetam could modulate the role of SV2A as an iTRAP, since it corrected synaptotagmin-1 levels in an SV2A overexpression system (Nowack et al., 2011). Adding further credence to a direct role for SV2A in epilepsy pathogenesis is the recent identification of a homozygous mutation (R383Q) in a highly conserved residue of SV2A in an individual with intractable epilepsy, developmental and growth delay (Serajee and Huq, 2015). It will be of great interest to determine how this mutation impacts on SV2A function and potentially synaptotagmin-1 trafficking.

Mice with a genetic ablation of both SV2A/B and stonin-2 display a worse seizure phenotype, and more prominent lethality than SV2A/B knockout mice alone (Kaempf et al., 2015) suggesting stonin-2 dysfunction may increase susceptibility to epilepsy. However stonin-2 knockout mice do not undergo seizures (Kononenko et al., 2013). Two exonic single-nucleotide polymorphisms in stonin-2 were associated with schizophrenia (Luan et al., 2011), however this finding could not be replicated in a separate study (Xiang et al., 2013). Instead, one of these polymorphisms (Ser307Pro) correlated with a larger cortical surface area on right inferior temporal hemisphere in individuals with schizophrenia (Xiang et al., 2013). The mechanisms by which this variant of stonin-2 causes this, as well as the functional relevance, remains unknown.

Thus, studies of human disease have provided tantalizing evidence that the potential dysfunction of the iTRAPs synaptophysin and SV2A have key roles in mammalian brain function. The fact that their roles become apparent in disorders that involve a fine balance between excitation and inhibition in complex neuronal circuits correlates with their emergence later in evolution, when fine control of neurotransmission is required to produce cognition, learning and behavior.

## The iTRAPs—Implications for Neuronal Function

The identification of the iTRAPs and their key role in ensuring the accurate retrieval of both sybII and synaptotagmin-1 during neuronal activity have revealed a hitherto unappreciated level of complexity in cargo selection and packing into SVs. The iTRAPS have appeared relatively late in evolution, with no close functionally-related homologs present in invertebrate species (Janz et al., 1998; Abraham et al., 2006; Yanay et al., 2008). This is in stark contrast to the monomeric adaptors, which are evolutionarily conserved from *C. elegans* and *Drosophila* through to higher order mammalian species. This suggests that as neuronal circuitry became more complex, additional mechanisms were required to ensure the efficient targeting and retrieval of essential SV cargo. The association of human neurological disorders with iTRAP dysfunction, as outlined above, adds further credence to the theory that the monomeric adaptors alone are not capable of maintaining the efficient retrieval of core SV proteins in complex mammalian circuits. The specificity of the iTRAPs in terms of their retrieval targets (sybII or synaptotagmin-1) is a key determinant of their function, with only very minor effects on SV turnover observed in either synaptophysin or SV2A knockout mice (Yao et al., 2010; Gordon et al., 2011; Kwon and Chapman, 2011). In contrast knockout systems for AP180/CALM and stonin-2 display more global defects in SV recycling. For example stonin-2 knockout neurons display alterations in both the kinetics and extent of CME and activity-dependent bulk endocytosis (Kononenko et al., 2013). Furthermore AP180 null organisms display an increase in SV diameter (Zhang et al., 1998; Nonet et al., 1999; Koo et al., 2011a, 2015; Petralia et al., 2013), a reduction in SV number (Bao et al., 2005; Petralia et al., 2013; Koo et al., 2015) and accumulation of endosomal intermediates in their nerve terminals (Koo et al., 2015).

The discovery of the iTRAP function of both synaptophysin and SV2A leads to the obvious question of whether any more iTRAPs exist. One potential candidate, as outlined above, is vGLUT, and it is a matter of urgency to determine whether it, or any other SV cargo (such as synaptoporin or synaptogyrin) perform similar functions. A critical area to address will be the relationship between the iTRAPs and the monomeric adaptor proteins that assist in retrieval of both sybII and synaptotagmin-1. The fact that depletion of both SV2A and stonin-2 resulted in an exacerbation of synaptotagmin-1 trafficking defects (Kaempf et al., 2015) suggests that they have complementary, rather than sequential functions. It will be of great interest to determine whether a similar relationship exists between synaptophysin and AP180/CALM. Another key question to determine is how the iTRAPs control the assembly of the retrieval complexes of either sybII or synaptotagmin-1 both temporally and spatially. This is of particular interest for SV2A, since a number of molecules share overlapping interaction sites for the C2B domain of synaptotagmin-1 (Leveque et al., 1992; Chapman et al., 1996, 1998; Schiavo et al., 1996; Bhalla et al., 2006). The phosphorylation-dependent control of the SV2A-synaptotagmin-1 interaction may be central to coordinating the hierarchy of association during endocytosis in this regard. Synaptophysin is the major vesicular phospho-tyrosine protein (Pang et al., 1988), and it will important to determine whether its binding to sybII is similarly regulated by phosphorylation.

The identification of the iTRAPs have revealed an additional layer of complexity in the retrieval of SV cargo during endocytosis. The specific roles of these proteins in coordinating the traffic of essential SV molecules, and their association with human neurological disorders, highlights the importance of having the correct complement of both sybII and synaptotagmin-1 on SVs.

## Author Contributions

SLG and MAC both devised and wrote this review article.

## Conflict of Interest Statement

The authors declare that the research was conducted in the absence of any commercial or financial relationships that could be construed as a potential conflict of interest.

## Abbreviations

iTRAP: intrinsic trafficking partner
SNARE: soluble NSF attachment protein receptor
SV: synaptic vesicle
CME: clathrin-mediated endocytosis
AP: adaptor protein
sybII: synaptobrevin II
CALM: clathrin assembly lymphoid myeloid leukemia
ANTH: AP-180 N-terminal homology
SV2A: synaptic vesicle protein 2A
vGLUT: vesicular glutamate transporter
AD: Alzheimer’s disease.
